# Early Appearance of Catamenia

**Published:** 1853-12

**Authors:** P. Van Patten

**Affiliations:** Walnut Camp, Arks.


### *Early appearance of the Catamenia.*

BY P. VAN PATTEN, M. D.

In offering the present paper for the criticism of the medical world, the writer is not unaware of the numerous objections and incredulous questionings which may be elicited; yet, multiform as they may prove —emanating from sources how high and respectable soever, considerations of professional obligation, vindicate its publicity. Youthful practitioners of our "art divine," are frequently obnoxious to the charge of discovering wonders which are marvelous and important to themselves alone—of agitating questions whose practical utility is inconsiderable—of predicating much upon conclusions far too precipitately deduced,—still a narration of the subjoined case is presented with little diffidence; the readers of the Journal may exercise their own judgment in its disposal, and if naught beside obtains, than a cursory perusal, no detraction will have been made from the peculiarities of the matter recorded; the facts relative, are before them *in extenso*.

During the month of July, ult., I was called to attend and prescribe for Mary E. P———, æt. 12 years. I found the patient laboring under intermittent fever, anæmic, debilitated, and possessing an enlarged spleen. While exhibiting the remedies indicated by her apparent condition, the mother remarked that she could acquaint me with facts relative to her daughter which to me, as a physician, might prove interesting. Manifesting a desire to be informed, she proceeded to state that her child had, during the preceding three or four months, been visited with a copious epistaxis, occurring at intervals of about four weeks each; that anterior to the first appearance of the epistaxis, she had menstruated regularly since the age of *three years*. The girl is of lymphatico-bilious temperament, diminutive in stature, and of medium muscular development; remarkable to the beholder, alone, for the dignified bearing and matronly conduct, as contrasted with her insignificant physical proportions. The external genitalia are large; no epidemic evidences of puberty present, the mons veneris and labia majora in that respect evincing adolescence. The mammæ have always been strikingly prominent. In color, fluidity, quantity, &c., the periodic flow has been quite uniform—each appearance preceded and accompanied by the sensations common to the catamenial juncture. About two weeks ago the menstrual molimen occurred as before; subsequently her health has improved rapidly.

In this instance, we have a case of premature special development co-existing with perfect functional power and action in an organism far removed from puberty; while the periodicity of the manifestations, together with the almost uniform health of the subject, demonstrate the presence of no abnormal condition. To aver the ova of such a child to be susceptible of impregnation, would be a labor of supererogation,—the legitimate presumption, however, is in favor of an affirmative opinion. *Qnery*: If the discharges regarded as menstrual, were *de facto*, not truly such, to what shall we refer them; were they evidences of disease?

Walnut Camp, Arks., Oct. 1853.

